# Peer Review of “Measuring Integrated Novel Dimensions in Neurodevelopmental and Stress-Related Mental Disorders (MIND-SET): Protocol for a Cross-sectional Comorbidity Study From a Research Domain Criteria Perspective”

**DOI:** 10.2196/36235

**Published:** 2022-03-29

**Authors:** Emma Palmer

**Affiliations:** 1 James L Winkle College of Pharmacy University of Cincinnati Cincinnati, OH United States

**Keywords:** psychiatry, mental health, psychiatric disorders, neuropsychology, stress, comorbidity

*This is a peer-review report submitted for the paper “Measuring Integrated Novel Dimensions in Neurodevelopmental and Stress-Related Mental Disorders (MIND-SET): Protocol for a Cross-sectional Comorbidity Study From a Research Domain Criteria Perspective.”*

## Round 1 Review

### General Comments

This paper [1] is interesting and sets the stage for a pretty comprehensive study.

### Specific Comments

#### Major Comments

1. The background is very long, and some spaces are redundant, talking about the overlap of symptoms in comorbidities. Some of this may be better in a discussion—there is a lot of information here.
2. There are a lot of definitive/overly positive statements (eg, “...the RDoC [Research Domain Criteria] frameworks fits ideally...” “...we can disentangle.” Consider rewording as this is a fairly small sample size in a singular area of the world.
3. Adjust the title so it is clear that this is a description of methods.
4. Anticipated limitations should be included (eg, single-center, nondiverse population, or the number of data points making differentiation challenging).

#### Minor Comments

1. Change addiction disorder to substance use disorder
2. Provide a citation for the first line about the acceptance of psychiatric comorbidities as common
3. Define abbreviations upon first use (eg, DSM-5)
4. Consider changing “healthy” to “neurotypical.” Personality traits were examined too, but there is not a lot of rationale here regarding overlap. I agree it is important to review this too, but this needs to be discussed.
5. Is microbiome included at the very end as a data point?

## Round 2 Review

It appears that the recommended revisions have been addressed, and I have no additional comments

## Abbreviations

RDoC: Research Domain Criteria

## References

[1] van Eijndhoven P, Collard R, Vrijsen J, Geurts DE, Vasquez AA, Schellekens A, van den Munckhof E, Brolsma S, Duyser F, Bergman A, van Oort J, Tendolkar I, Schene A (2022). Measuring Integrated Novel Dimensions in Neurodevelopmental and Stress-Related Mental Disorders (MIND-SET): protocol for a cross-sectional comorbidity study from a Research Domain Criteria perspective. JMIRx Med.

